# Peritoneal Dialysis Versus Extracorporeal Ultrafiltration Modalities in the Management of Acute Cardiorenal Syndrome with Diuretic Resistance

**DOI:** 10.3390/life16020249

**Published:** 2026-02-02

**Authors:** Laura Elena Zamora-Cervantes, Enzo Vásquez-Jiménez, José Manuel Rodríguez-Chagolla, Mayra Eugenia Avilés-Ramírez, Viridiana Galicia Galicia, Roberto Galindo-López, Alberto Ramírez-Gil, Diego Sánchez-Hernández, Octavio René García-Flores, Hiram José Serrano-García

**Affiliations:** 1 Department of Nephrology, Hospital Juárez de México, Av Instituto Politécnico Nacional 5160, Magdalena de las Salinas, Gustavo A. Madero, Mexico City 07760, Mexico; lolezamora@gmail.com (L.E.Z.-C.); maeu21.aviles@gmail.com (M.E.A.-R.); viri.galicia.galicia@gmail.com (V.G.G.); robrtogl1@gmail.com (R.G.-L.); alb_rg@hotmail.com (A.R.-G.); sahendiego@gmail.com (D.S.-H.); garoct15230@gmail.com (O.R.G.-F.); hjso4@hotmail.com (H.J.S.-G.); 2 Department of Nephrology, Centro Médico ISSEMYM, Toluca 52170, Mexico; jm.rodriguez.chagolla@gmail.com

**Keywords:** acute cardiorenal syndrome, diuretic resistance, peritoneal dialysis, extracorporeal ultrafiltration, congestion, diuretics

## Abstract

In cardiorenal Syndrome (CRS), diuretic resistance is frequent and congestion predominates in acute forms. In refractory cases, non-pharmacological ultrafiltration therapies have shown effectiveness on fluid removal, improved diuresis, and reduced rehospitalizations. However, the choice of modality remains individualized and resource-dependent. Both continuous renal replacement therapy (CRRT) and peritoneal dialysis (PD) offer hemodynamic advantages, but CRRT carries risks such as infection, bleeding, and high cost. PD has also demonstrated benefits in acute settings, providing effective sodium removal, and no need for anticoagulation, though it is not considered first-line therapy. There are few studies comparing different renal replacement therapies (RRT) in patients with acute CRS and there is no evidence on diuretic resistance. So, the question arises: could there be an advantage of a modality beyond fluid removal? The scarcity of comparative studies underscores the need for randomized trials that move beyond the cardiocentric perspective and include patients at risk of diuretic resistance.

## 1. Introduction

Which therapy should be chosen once there is diuretic resistance in acute cardiorenal syndrome? Congestion is a frequent, though not exclusive, hallmark of acute CRS [1]. The effectiveness of diuretics is often limited in CRS, leading to diuretic resistance [2]. Even non-pharmacological ultrafiltration therapies have proven effective in volume control [3]; the selection between them depends on the hemodynamic profile and availability of the centers. While CRRT is mainly used in acute and unstable forms of CRS, it carries risks such as infection, bleeding, and high cost. PD is reserved for chronic forms and frail patients and has also demonstrated benefits in acute settings, providing effective sodium removal, and no need for anticoagulation, though it is not considered first-line therapy. Could the choice depend on an advantage offered by a modality beyond fluid extraction? The purpose of this opinion is to highlight the current evidence on ultrafiltration therapies, with the aim of comparing PD and extracorporeal therapies.

### 1.1. Cardiorenal Syndrome

Cardiorenal syndrome comprises a spectrum of disorders involving the heart and kidneys, in which one induces dysfunction of the other, either acutely and/or chronically [4]. Two major groups are recognized according to the trigger in the pathological process: cardiorenal and renocardiac [5]. Traditionally, five phenotypes are identified based on the duration of the condition: two acute, two chronic, and one mixed systemic [6].

Acute CRS (type 1), decompensated heart failure (HF) that results in acute kidney injury (AKI), which has been defined as creatinine ≥ 0.3 mg/dL in 48 h or uresis < 0.5 mL/kg/h for 6 h [7], has a prevalence that can vary from 10% to 40%, which depends on the different definitions of AKI [8]; although frequently present with congestion [1], it is also present in the chronic forms and in acute renocardiac syndrome. Since kidneys are the main effectors in the activation of the neurohormonal system, they play a key role in congestion associated with CRS. In chronic heart disease, up to 30–50% of patients show an alteration in kidney function (type 2 CRS), where congestion is also a key factor in decompensation [9]. While in type 4 CRS—chronic kidney disease (CKD) with HF—the prevalence of congestion is especially serious, reaching 60%, with 14% of the patients in hemodialysis suffering one or more episodes of acute pulmonary edema over a period of 2 years [10].

Patients with acute or chronic CRS have a worse prognosis, with significantly prolonged hospitalization, associated with a higher risk of rehospitalization and increased mortality [11].

Across all CRS phenotypes, prognosis remains poor. Patients with acute or chronic CRS experience longer hospital stays, higher rates of rehospitalization, and increased mortality [11]. A meta-analysis reported a higher prevalence of CRS among patients with heart failure or acute coronary syndromes (32%; 95% CI 0.23–0.41) compared with those presenting primarily with AKI (15%; 95% CI 0.02–0.29), with an overall CRS-related mortality of 28% (95% CI 0.18–0.35) [12]. Importantly, acute CRS events carry a particularly adverse prognostic impact, with a markedly increased risk of death in type 1 and type 3 CRS compared with chronic type 4 CRS [13]. Even among patients with established chronic kidney disease, superimposed acute events such as cardiogenic shock or acute rises in creatinine at hospital admission are associated with a significant increase in short-term mortality and adverse cardiac outcomes [14,15].

### 1.2. Congestion in Cardiorenal Syndrome

Congestion is a hallmark of cardiorenal syndrome and a major determinant of prognosis. It is traditionally defined as extracellular fluid accumulation secondary to increased cardiac filling pressures, altered venous compliance, and plasma volume expansion [16]. From a clinical perspective, however, congestion in CRS is better understood as a dynamic process driven predominantly by renal sodium avidity and sustained neurohormonal activation rather than by excess free water alone [17].

Persistent congestion is associated with adverse outcomes, including prolonged acute kidney injury, increased need for renal replacement therapy, and higher mortality [18]. Importantly, congestion encompasses distinct but overlapping phenotypes—intravascular, interstitial (tissue), and pulmonary—which differ in pathophysiology and therapeutic response [19]. Intravascular congestion is characterized by neurohormonal activation and increased proximal tubular sodium reabsorption, leading to plasma volume expansion and elevated venous pressures [20,21]. Interstitial congestion develops through progressive sodium accumulation within the extracellular matrix, lowering resistance to fluid expansion and promoting edema even with modest increases in hydrostatic pressure [22]. Pulmonary congestion may result from rapid blood volume redistribution without proportional increases in total body water [23,24].

All mechanisms increase venous return, cardiac preload, and cardiac filling pressures. As intravascular congestion starts, hydrostatic pressure increases both intraglomerular capillaries and in the interstitium. This leads to renal hypoperfusion and promotes a vicious cycle that may ultimately lead to diuretic resistance [25].

### 1.3. Diuretic Resistance in Cardiorenal Syndrome

Loop diuretics have proven their efficacy, especially in decompensated HF, since they facilitate rapid decongestion and symptom relief; however, diuretic resistance is still a challenge within the CRS context, which may occur in up to 50% [26], since it is associated with an adverse prognosis, greater mortality, and higher treatment costs [27].

Even if there is no clear definition for diuretic resistance, it is understood as a reduced sensitivity to diuretics, despite the gradual increase in loop diuretic doses, which results in a decrease in natriuresis [28] (urinary sodium below 70 mmol/L) and diuresis (urinary volume below 150 mL/h), limiting the possibility of reaching euvolemia [29].

Diuretic resistance is explained by the interaction between extratubular and intratubular mechanisms. Renal perfusion and renal venous congestion, in addition to tubular adaptation, secondary to neurohormonal activation and prior exposure to diuretics and natriuretic peptide expression, shift the loop diuretic response curve downward and to the right, requiring higher and sequential doses, yet resulting in reduced natriuresis [30,31]. In addition, hypertrophy of distal tubular cells increases the expression of sodium transporters and vasopressin-mediated free water retention, promoting sodium and water reabsorption, which reinforces a state of functional resistance [32,33,34].

### 1.4. Current Therapies Against Diuretic Resistance

In order to mitigate marked sodium retention and reduced natriuresis, the use of frequent doses or continuous infusion of a loop diuretic is required, which may be up to 10 to 15 mg/h. The DOSE-HF trial showed that a greater diuresis (3575 mL vs. 4899 mL, *p* < 0.001) [35] and early transition to oral diuretics (31% vs. 17%, *p* < 0.001) [36] were achieved at high doses of furosemide (2.5 times higher). Therefore, initial doses of 1 mg/kg or up to 80 mg are recommended if estimated glomerular filtration rates are below 60 mL/min/1.73 m^2^, and 1.5 mg/kg or twice the dose in cases of prior use, in order to prevent resistance. Continuous infusion has a theoretical advantage in the case of a slightly higher weight loss (−1.08 kg; 95% CI −1.39; −0.8) [37].

In the case of an inadequate response, once the loop diuretic dose has been optimized, sequential blockade is indicated [36]. The addition of acetazolamide (an inhibitor or carbonic anhydrase) in the proximal tubule showed benefits in the ADVOR trial, with a greater clinical decongestion (42.2% vs. 30.5%; RR 1.46; *p* < 0.001) and greater diuresis after 48 h, with no impact on mortality or rehospitalizations [38]. SGLT2 inhibitors induce a mild and transient natriuresis; in the EMPULSE trial, empagliflozin increased weight loss (−1.97 kg; *p* < 0.0001) [39], while in the DICTATE-AHF [40] and DAPA-RESIST trials [41] no significant differences in weight were observed, nor mortality at 90 days (*p* = 0.589) [41], although a reduced accumulated dose of furosemide was observed (560 mg vs. 800 mg, *p* = 0.006) [40].

Thiazides, by blocking distal reabsorption, enhance the effect of loop diuretics, as proven by CLOROTIC with a greater diuresis (1775 vs. 1400 mL; *p* = 0.05) and weight loss (−2.3 vs. −1.5 kg; *p* = 0.002), even in advanced CKD; when added to furosemide, a greater fluid loss was observed (−0.4; 95% CI −0.2 to −0.7; *p* < 0.001), but also no differences in mortality at 30 or 90 days were observed [42]. In contrast, aldosterone antagonists, such as spironolactone, do not improve acute decongestion (ATHENA-HF), although they reduce mortality and hospitalizations in chronic HF [43]. Finally, aquaretics (tolvaptan and hypertonic solution) increase diuresis and reduce weight, without survival benefits, and are reserved for specific cases, such as hyponatremia or advanced kidney disease [44].

Despite available pharmacological strategies, a patient subgroup with refractory congestion persists. In these cases, ultrafiltration (UF) therapies, such as peritoneal dialysis and hemodialysis, arise as effective alternatives for volume control. There is substantial evidence that compares UF to diuretic treatment, but the results are still controversial. No differences in weight loss were observed in the CARRESS-HF trial (ultrafiltration in decompensated heart failure with cardiorenal syndrome) between both groups (*p* = 0.87), but there was a significant decrease in creatinine in the UF group (*p* = 0.003), although 60-day mortality was similar between groups (*p* = 0.47) [45]. In contrast, in the UNLOAD trial, UF was associated with a greater weight loss (5.0 vs. 3.1 kg, *p* = 0.001) and less rehospitalization at 90 days (*p* = 0.037) [46]. Also, UF was associated with an increase in uresis (198 mL vs. 61.77, *p* < 0.001) [47]. Finally, a meta-analysis showed the advantage of UF for fluid elimination, with a difference of 1372.5 mL (95% CI 849.6 mL to 1895.4 mL; *p* < 0.001, I^2^ = 48.41%) [48].

The association of UF with reduction in rehospitalizations due to HF exacerbation compared to diuretics according to a meta-analysis is an (OR 0.543; 95% CI 0.362 to 0.815; *p* = 0.003, I^2^ = 68.4%) and a lower risk of decompensation (OR 0.63; 95%CI 0.43 to 0.94; *p* = 0.022) [48]; however, mortality with UF is not significantly different compared to diuretics [49]. Furthermore, different etiologies among patients treated with diuretics vs. UF, together with adverse events associated with UF, do not provide sufficient evidence to prove that UF is better.

Then, the real impact is about weight loss or congestion. When studies compare UF or diuretics, decongestion is defined by fluid removal and weight loss; however, congestion is more than fluid accumulation, but the balance between intravascular and interstitial space, so free water removal will not be enough.

From our perspective, congestion cannot be understood without recognizing the central role of sodium, as it depends on the interaction between the extracellular and intracellular spaces. In this context, true decongestion does not depend on the elimination of free water but on the effective removal of sodium, where diuretics play a key role. This was supported by the ROSE-AHF trial, where sodium excretion was associated with lower mortality [50]. Furthermore, extracorporeal replacement therapies (intermittent and continuous) and peritoneal dialysis perform more than just UF of free water in cases of congestion. However, the available evidence does not demonstrate a clear difference in outcomes between the different modalities, which suggests that the choice of treatment should be individualized according to the patient’s hemodynamic profile, comorbidity, and congestion status.

### 1.5. Intermittent or Continuous Extracorporeal Ultrafiltration vs. Peritoneal Dialysis

#### 1.5.1. Intermittent

Intermittent UF can be combined with diffusion for the removal of small solutes or convection for elimination of medium-sized molecules. It is applied for short periods of time (3 to 5 h), conditioning the UF rate to the patient’s hemodynamic tolerance [51].

#### 1.5.2. Hybrid

It combines the diffusion and convection principles—whose subtypes are prolonged dialysis, slow low-efficiency dialysis, and prolonged intermittent dialysis—differentiating itself because it is administered during longer periods of time than intermittent treatments (6 to 12 h, compared to 3 to 4 h, respectively) but shorter than the continuous ones. They may be performed through intermittent hemodialysis machines or CRRT machines [52].

#### 1.5.3. Continuous Renal Replacement Therapy

It is mainly based on the convection and diffusion principle, and it is applied slowly and continuously 24 h per day [53]. It allows solute clearance in case of acidosis, hyperkalemia or uremia, and offers the possibility of isolated UF in order to optimize fluid extraction and eliminating sodium, with a better hemodynamic tolerance by keeping a constant volume, due to the balance between interstitial and intravascular space filling within the context of CRS [54].

The evidence on the indication of intermittent versus continuous renal replacement therapy in CRS regarding the acute type is controversial. Even in critical patients, no difference has been observed in hospital mortality (62.5% vs 58.1% for intermittent and CRRT, respectively; *p* = 0.43) [55]. Although CRRT offers more hemodynamic stability and allows combining convective, diffusive, and UF modalities [56], in comparative studies focused on acute CRS, mortality is still high—30% to 70% per year [57,58]. It is important to consider that the high burden of comorbidities and the presence of shock may influence these outcomes.

#### 1.5.4. Peritoneal Dialysis

In PD, fluid is instilled into the peritoneal cavity, which allows a continuous and effective elimination of solutes, including sodium and potassium, through the principle of diffusion driven by the concentration gradient between the capillaries and the dialysate. UF occurs due to the osmotic force created by hypertonic dialysate (glucose at 1.5%, 2.5%, or 4.25%), which generates a transmembrane pressure gradient [59].

The modalities suggested for patients with CRS are adapted from those intended for chronic HF [60]. These have been continuous ambulatory dialysis or automated dialysis, with doses according to residual uresis and the UF requirement; in the first case, with a daytime icodextrin exchange, or at 4.25%, and nighttime dry cavity; or two daytime 2.5% exchanges and one 2.5% nighttime exchange; or two to three nighttime exchanges with a 2.5% solution. In the case of minimal residual uresis, with a daytime dose of two to three exchanges with 2.5%, and nighttime with one 2.5% exchange, icodextrin or 4.25% [61].

In acute patients—even those without CRS but with acute kidney injury—in order to minimize acute complications after the insertion of the catheter within 48 h. Based on a trial, the suggestion is to initiate volumes according to the body surface area (BSA): 750 mL for BSA < 1.7 m^2^ and 1000 mL for BSA > 1.7 m^2^ with volume increasing on day 7; the complications involving leakage were observed in 5% [62]. Studies targeting patients with acute CRS have initiated 1500 mL after insertion and adjusted it after 3 days depending on volume status and metabolic profiles [60]. Additionally, with a low volume of 750 to 1000 mL, with a minimum of two to three exchanges or icodextrin for UF purposes [63].

PD may offer advantages by avoiding activation of the sympathetic nervous system and the renin–angiotensin–aldosterone axis which, as opposed to extracorporeal UF, allows a more stable extraction of volume, reducing the risk of myocardial stunning [64]. In addition, it reestablishes the diuretic response through the removal of sodium and water [65], which has been corroborated with sodium removal therapies through the peritoneal membrane (Alfapump DSR system and Celsite T203J), which showed a significant increase in the diuretic response (81 to 233 mmol, *p* = < 0.001) and an improvement in markers such as CA-125 and pro-BNP (*p* < 0.003) [66].

It has even been proposed as an adjuvant to SGLT2 inhibitors to enhance the diuretic and natriuretic response, since the peritoneal membrane also expresses SGLT2 cotransporters [67].

Various trials have shown the results of PD in CRS, observing safety in acute patients, without a significant association between catheter dysfunction or peritonitis and mortality (*p* = 0.06 and *p* = 0.63, respectively) [68]. Also, according to a meta-analysis that included 29 observational trials in patients with chronic HF, nine of them with type 2 CRS treated with PD showed a significant improvement of the LVEF (3.67%, 95% CI 0.80 to 6.53%, *p* = < 0.01; I^2^ 74.5%) [69] and a reduction in annual hospital stay (−34.8 days, 95% CI −20.3 to 48.9, *p* < 0.0001; I^2^ 92%) [69]. Limitations can be observed when starting a patient on PD, since it requires experience for insertion, as well as center organization for personal training to ensure UF [70].

Currently available studies on PD in chronic CRS have shown a 1-year survival of 85.2% to 66%, although higher cardiovascular mortality compared to non-CRS patients; higher prevalence of comorbidities, such as age, fragility, and more severe cardiac disease should be noted [71,72].

Likewise, PD is associated with better preservation of residual renal function, contributing to the excretion of phosphorus and the control of mineral balance; from an endothelial perspective, the combination of continuous purification, less inflammation, and lower variability of phosphorus could have the potential to mitigate vascular damage associated with phosphorus and left ventricular hypertrophy in CRS [73].

Despite the above, comparative evidence between PD and extracorporeal therapies is mainly from retrospective cohorts, reviews, and randomized trials that are not specifically focused on patients with cardiorenal syndrome (Table 1).

While both PD and extracorporeal UF has proven to be safe and effective to net fluid loss, most of the trials maintain a cardiocentric approach, focusing on decompensated HF, without evaluating in a comprehensive way the deterioration of the function, or renal outcomes; likewise, only a few trials explicitly address diuretic resistance, and a survival benefit has not been clearly established, nor are significant differences shown, such as in hospital stay or quality of life.

## 2. Future Perspectives

It is suggested to continue with future lines of research focused on randomized clinical trials that directly compare the different UF strategies in patients with cardiorenal syndrome, incorporating renal and cardiac outcomes beyond cardiac decompensation. It will be essential to explore biomarkers together with congestion phenotyping that predict the diuretic response and assess the role of combination therapies—in order to optimize decongestion and preserve renal function.

## 3. Conclusions

In cardiorenal syndrome, diuretic resistance is frequent and congestion predominates in the acute forms, although it may also appear in chronic forms. In refractory congestion, non-pharmacological UF therapies have been associated with more fluid removal, improvement in diuresis, and a decrease in rehospitalizations; however, the choice of modality continues to be individualized and dependent on the available resources. Even if continuous renal replacement therapy has hemodynamic advantages, its use requires assessing potential complications, such as infections, bleeding, and high associated mortality, which could be related to the optimal timing of initiation, which remains unknown. Although the current studies do not include CRS, fluid removal seems to be the main indication [58]. Peritoneal dialysis has been well established in chronic cardiorenal syndrome, has also shown benefits in acute scenarios—since it does not require anticoagulation—and maintains hemodynamic stability and efficacy in sodium removal, although there are still reservations about considering it as a first-line option. However, in the long term, an improvement in mortality in favor of peritoneal dialysis can be observed. Overall, the scarce comparative evidence between UF modalities underscores the need for randomized clinical trials with a comprehensive approach that goes beyond the current cardiocentric perspective and includes patients with diuretic resistance risk, especially those with chronic cardiorenal syndrome that allows us to individualize therapies.

## Figures and Tables

**Table 1:** Evidence comparing peritoneal dialysis versus extracorporeal therapies in cardiorenal syndrome (Abbreviations: HF, heart failure; HD, hemodialysis; PD, peritoneal dialysis; APD, automated peritoneal dialysis; CRS; cardiorenal syndrome; CHF, chronic heart failure; CKD, chronic kidney disease; SBP, systolic blood pressure; HR, Hazard ratio; UF, ultrafiltration; TPD, tidal peritoneal dialysis; LVEF, left ventricular ejection fraction; CVVHDF, continuous veno-venous hemodiafiltration; AKI, acute kidney injury; ICU, intensive care unit; KRT, kidney replacement therapy; RKF, recovery of kidney function; OR, odds ratio; VA-ECMO, Venoarterial extracorporeal membrane oxygenation; AHF, acute heart failure.

Study	Modality	Description	Population	Results	Conclusion	References
Congestive HF treated with PD or HD retrospective cohort study (2021)	PD (45%)/APD (26%) vs. HD 3/week (93%) HD 4/week (4%)	Retrospective cohort study	Chronic CRS, CHF + CKD *n* = 264	SBP HR 0.95 (95% CI 0.93–0.97, *p* < 0.0001) 1-year mortality HR 1.44 (95% CI 0.68–3.03, *p* = 0.35) 2-year mortality HR 1.69 (95% CI 0.92–3.12, *p* = 0.1)	No modality associated with higher risk of death despite different patient profile	[74]
TPD versus UF in CRS 1: A Prospective Randomized Study (2019)	TPD (*n* = 44): 20–25 L/day, filling volume of 1.5–2 L for 90–120 min dwell time, 12–14 cycles/day UF (*n* = 44): blood flow rate 100–170 mL/min, UF rate 75–120 mL/h	Randomized clinical trial 1:1	Acute CRS *n* = 88	Net fluid loss in TPD 5.5 kg vs. 3.1 kg in UF (*p* = 0.018) Change in LVEF 47% in TPD vs. 34% in UF at 72 h (*p* = 0.044) and 50% vs. 36% at 120 h (*p* = 0.032) More episodes of hypotension, arrhythmias, infections and catheter malfunction in the UF group	TPD was associated with improvement of LVEF and net fluid loss compared to UF with less adverse events	[75]
Acute Kidney Injury in Critically ill patients: A Prospective Randomized Study of Tidal Peritoneal Dialysis versus Continuous Renal Replacement Therapy (2018)	CVVHDF (*n* = 62): blood flow rate 180–200 mL/min, effluent rate 30 mL/kg/h, TPD (*n* = 63): 25 L/day, filling volume 2 L, tidal volume 70%, total 24 h	Randomized clinical trial 1:1	AKI in ICU, 7.2% with acute CRS (CVVHDF 8.1%, TPD 6.3%) *n* = 125	RKF in TPD vs. CVVHDF improved 60.3% vs. 35.5% (*p* = 0.005) Resolution of AKI in TPD vs. CVVHDF at 5 days vs. 8 (*p* = 0.004) More complications related to CVVHD: hypotension (*p* = 0.0016), infections (*p* = 0.003), arrhythmias (*p* = 0.0023) Mortality with CVVHDF OR 0.79 (95% CI 0.68–0.11, *p* = 0.001)	Better outcomes with TPD compared to CRRT, but CVVHDF has a lower association with mortality; however, the heterogeneity of AKI etiology and the proportion of CRS patients should be considered.	[76]
Outcomes of Continuous renal Replacement Therapy versus peritoneal dialysis as a renal Replacement Therapy modality in patients undergoing Venoarterial extracorporeal membrane oxygenation (2024)	CRRT (*n* = 21): 30.2 mL/kg/h, blood flow rate 170.5 ± 29.2 mL/min PD (*n* = 22): 15 solution, dwell volume 1000 mL/h, 24 L/day (Selection of modality was based on the physician’s preference)	Retrospective study	AKI in patients underwent VA-ECMO Acute CRS *n* = 43	Mortality rate of 80.9% for CRRT and 90.9% for PD (*p* = 0.35) Total UF in CRRT 3580 mL vs. 10050 mL in PD (*p* = 0.0036) Urine output during the first 3 days in CRRT was 1045 vs. 288 in PD (*p* = 0.0321)	No significant difference in hospital mortality between CRRT and PD in VA-ECMO patients requiring CRRT, but PD demonstrated higher UF rates and more negative fluid balance compared to CRRT	[77]
Cardiac Surgery Outcomes in Patients Receiving Hemodialysis Versus Peritoneal Dialysis (2023)	PD (11%) Intermittent or continuous HD (89%)	Retrospective study	Chronic CRS CKD on KRT+ AHF *n* = 590	Survival after 30 days in PD was 95.7% and HD 98.2% (*p* = 0.30) Length of stay 10 for PD vs. 11 (*p* = 0.21) Composite events such as effusion free and external infection free were not significant either (*p* = 0.05 and *p* = 0.32)	PD compared with HD does not appear to improve outcomes of patients with CKD undergoing cardiothoracic surgery	[78]

## Data Availability

No new data were created or analyzed in this study. Data sharing is not applicable to this article.
